# Characteristics of the Competency Ethical Principles for the Nurse Manager: A Systematic Review

**DOI:** 10.1155/jonm/2575609

**Published:** 2025-02-26

**Authors:** Alberto González-García, Arrate Pinto-Carral, Pilar Marqués-Sánchez, Cristina Liebana-Presa, Rubén García-Fernández, Silvia Pérez-González

**Affiliations:** ^1^Faculty of Health Sciences, Nursing and Physiotherapy Department, Universidad de León, León 24007, Spain; ^2^Faculty of Health Sciences, Nursing and Physiotherapy Department, SALBIS Research Group, Campus de Ponferrada, Universidad de León, León 24402, Spain

## Abstract

**Background:** The escalating complexity of healthcare environments underscores the importance of nurse managers' ethical competency, which impacts organizational culture, work climate, and healthcare outcomes.

**Objective:** The purposes of this study were to identify and describe the characteristics of nurse managers' competency in ethical principles in healthcare settings.

**Methods:** A systematic search was conducted across the Web of Science, Scopus, and PubMed databases, covering the literature from January 1, 2014, to April 1, 2024. Articles were selected based on specific inclusion criteria, and data were extracted and analyzed using a frequency analysis of the identified characteristics. This systematic review adhered to the PRISMA guidelines and the Joanna Briggs Institute assessment tools. The review protocol was registered in PROSPERO (ID: CRD42024542594).

**Results:** The review analyzed 22 studies, each focusing on nurse managers and their ethical competencies. Fourteen distinct characteristics of ethical competency were identified, with ethical leadership, ethical decision-making, and conflict resolution being the most prominent. Key ethical problems included conflicts between personal values and organizational policies, pressures to compromise ethical standards, and management of limited resources. These problems often led to significant consequences such as decreased staff morale, increased stress and burnout, and compromised patient care quality.

**Conclusions:** The development of ethical leadership and the ability to make ethical decisions are fundamental for nurse managers to create supportive work environments and reduce burnout. Promoting continuous professional development and fostering an ethical culture is essential for maintaining high ethical standards. Future research should explore how cultural, organizational, and environmental differences influence ethical decision making and leadership.

**Implications for the Nursing Management:** The development of ethical leadership and the ability to make ethical decisions are fundamental characteristics of ethical principles competency, as they facilitate the achievement of an adequate work environment and reduce burnout and emotional overload.

## 1. Background

The healthcare sector is one of the most significant sectors worldwide due to its extensive scale and the social and economic implications [1–3]. Moreover, this sector is increasingly complex and marked by continuous technological advancements that complicate the interactions and safety of both individuals and professionals [4–6]. The current healthcare environment emphasizes two crucial dimensions of social judgment: competence, associated with skills and intelligence, and warmth, involving morality and sociability, pivotal in shaping the perception of others as opportunities or threats [7–10].

Ethics in work environments are considered a driving force behind economic activity at all societal levels [11]. Ethics, the branch of philosophy concerned with moral life aspects and norms that are widely and stably accepted in society, is crucial [12, 13]. Ethical principles are broad statements that translate conceptual beliefs into ethical [14, 15]. Ethics are manifested in the relationships that individuals, groups, or organizations establish, becoming particularly significant in intense contexts such as those within healthcare organizations [16–18].

Few examples are closely linked to ethics as the ethics of care, which is fundamental at theoretical, philosophical, and practical levels and is the key to nursing [19, 20]. The nurse manager is responsible for planning and managing resources, organizing nursing teams, supporting teamwork, evaluating the services provided, and contributing to the achievement of outcomes. They are also responsible for resolving ethical dilemmas related to care in healthcare organizations, fostering an ethical culture, and leading ethical values [21–25]. García et al. [23] included ethical principles as a key competency for nurse managers.

In this sense, the literature has documented that the development of competencies alone is not sufficient to determine the success of managerial roles. In fact, competency also refers to unobservable human factors such as attitudes, individual attributes, and values [26]. Competency in emotional intelligence, integrity, ethical principles, and human warmth is also necessary, with special attention to moral issues [27]. Therefore, we ask ourselves, “What are the characteristics of ethical principles competency?” This systematic review aims to describe the characteristics of the competency of ethical principles. In addition, we set out to describe the main ethical problems faced by nurse managers, identify the primary consequences of these problems on healthcare environments, and describe the application of key ethical principles by nurse managers in addressing such problems.

## 2. Methods

### 2.1. Design

A systematic review was conducted to identify and describe the characteristics of nurse managers' ethical principles competency. This review adhered to the Preferred Reporting Items for Systematic Reviews and Meta-Analyses (PRISMA) guidelines and checklists [28, 29]. The Joanna Briggs Institute (JBI) guidelines were used to assess the quality of the included articles [30, 31]. The research question, formulated using the Population-Concept-Context (PCC) framework, was as follows [32]: What are the characteristics (C) of nurse managers' ethical principles competency (P) in the healthcare setting (C)? The review protocol was registered in PROSPERO (ID: CRD42024542594).

This study replicated the methodology established by González-García et al. [27] by incorporating a search strategy, article selection criteria, and data extraction and coding procedures. This methodology was selected because the current research aligns with the ongoing line of inquiry that aims to implement a competency model for nurse managers, developed by González-García in 2019 [33]. Ensuring methodological consistency is crucial for maintaining the coherence of research findings. In addition, this process is registered in the intellectual property registry 00/2024/3056 of the Ministry of Culture in Spain.

### 2.2. Search Strategy

A systematic search was conducted across three databases: Web of Science, Scopus, and PubMed, covering the period from January 1, 2014, to April 1, 2024. This timeframe was selected to gather the most current evidence. The keywords used were those described by González-García [33], consistent with the methodological strategy of the research line. These terms included “nurse manager,” “nurse supervisor,” “nursing program manager,” “nurse unit manager,” “chief nurse executive,” “nurse administrator,” “director of nursing,” “head nurse,” “frontline manager,” “nursing director,” “nursing executive,” and terms directly related to the ethical principles competency within the same research framework. The search strategy used are listed in Table S1 and Supporting File 1.

### 2.3. Inclusion and Exclusion Criteria

Quantitative, qualitative, and mixed methods articles addressing ethical principles related to nurse management, published between January 1, 2014, and April 1, 2024, were included. Articles lacking focus on nurse management or ethical principles pertinent to this field were excluded. Review articles and research that had not undergone peer review were also omitted.

### 2.4. Study Selection

The selection process entailed three main steps: (1) the search results were initially exported to Mendeley and subsequently transferred to Covidence; (2) duplicates and nonrelevant articles were removed through a thorough screening of titles and abstracts; and (3) articles were excluded in accordance with the established eligibility criteria. The screening of titles, abstracts, and full texts of each retrieved article was independently conducted by two researchers (AG and PM). Any disagreements between the researchers were resolved through discussion and, if necessary, further reviewed by a third researcher (SP).

### 2.5. Data Extraction

The initial search was conducted by a member of the research team responsible for eliminating duplicates and assessing titles and abstracts. Articles that appeared relevant to identifying specific characteristics were selected for an independent review by two other team members. Subsequently, articles that met the inclusion criteria were further examined by four team members who determined which studies would be incorporated into this systematic review.

Data collection was conducted using specially designed forms, which facilitated the gathering of information on the study type, sample size, participant characteristics, countries involved, and other attributes relevant to ethical principles. Data analyzes were performed using Microsoft Excel. For organization and classification, a specific coding system was implemented: each article was assigned a unique identifier starting with the letter “A” followed by three digits, beginning with A001 for the first article analyzed. Similarly, each identified characteristic received a code starting with “C,” followed by three digits starting with C001. For instance, “A003 C007” denotes the seventh characteristic identified in the third article. Identical characteristics were collectively analyzed, and the frequency of occurrence was recorded. In this review, the term “ethical principles characteristic” specifically denotes an attribute related to the ethical principles and competence of nurse managers.

### 2.6. Quality Appraisal

Studies included in this review were independently evaluated using the JBI tools [30, 31]. These tools include various items that evaluate the alignment of the research methodology with the research question, data collection, analysis methods, researcher roles, and cultural considerations. Responses to these items are categorized as “yes,” “no,” “uncertain,” or “not applicable.” The risk of bias in individual studies was assessed at the study level using the JBI Critical Appraisal Tools for both quantitative and qualitative studies. The risk of bias informed the narrative synthesis, categorizing studies as low, moderate, or high risk, and guiding the weighting of evidence. Furthermore, a qualitative sensitivity analysis was performed by excluding high-risk studies to assess the potential impact on the review's conclusions. The quality assessment was conducted independently by two team members (AG and PM), and any discrepancies were resolved through team discussion and consensus.

### 2.7. Data Analysis and Synthesis

The potential for conducting a meta-analysis was evaluated; however, due to the heterogeneity of the data, we opted instead for a frequency analysis of the identified characteristics and a contextual analysis of ethical principles competency. Each article included in the review was thoroughly examined to identify the following: (1) author and year of publication, (2) study design, (3) variables studied, (4) methodology used in the management of ethical principles, (5) nurse managers' approaches to ethical principles, and (6) the quality assessment of the study. In addition, the SWIM methodology was adapted to suit the specific needs of this study [34]. This adaptation included several steps: (1) summarizing articles, their results, and methodological quality; (2) identifying similar studies for comparison and pooling; (3) selecting data relevant to the study objectives; and (4) synthesizing the evidence coherently to formulate conclusions that align with our research objectives.

## 3. Results

**Figure 1:**
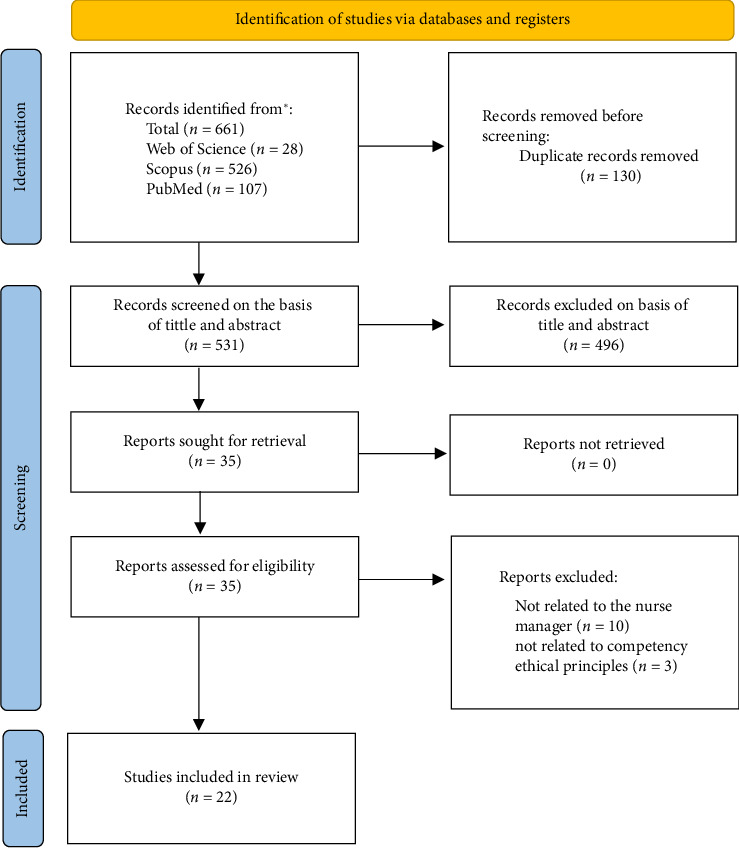
PRISMA 2020 flow diagram.

This systematic review included 22 studies that analyzed the characteristics of ethical principles competency in nurse managers (Table S2 and Supporting File 1). The study selection was conducted through an exhaustive search of the Web of Science, Scopus, and PubMed databases. From the initial 661 records, 130 duplicates were removed and 531 studies were selected based on their titles and abstracts. Of these, 496 were excluded and 35 full-text studies were assessed for eligibility. Ultimately, 22 studies met the inclusion criteria and were analyzed in detail (Figure 1).

The articles included in the review provide a comprehensive overview of the main ethical dilemmas faced by nurse managers, including those related to personnel management, organizational challenges, and the need for ethical decision-making.

The results of the quality analysis ranged from moderate to high. Quantitative studies showed high adherence to the quality criteria, with scores ranging from 87.5% to 100%. These scores indicated a reliable level of methodological consistency (Table S3 and Supporting File 1).

In contrast, the qualitative studies also demonstrated high quality, with scores ranging from 80% to 100% (Table S4 and Supporting File 1). Lower scores, mainly observed in some qualitative studies, suggest possible discrepancies with the JBI qualitative assessment tool. Nevertheless, the inclusion of these studies was considered essential to broaden our understanding of the ethical dilemmas in nursing management.

The categorization of the studies based on their risk of bias (low, moderate, and high) guided the weighting of evidence in the narrative synthesis. In our investigation, 4% of the studies were classified as low risk, 91% as moderate risk, and 5% as high risk. This distribution highlights the predominance of studies with moderate risk and underscores the need for a careful interpretation of the findings of high-risk studies (Table S5 and Supporting File 1).

Regarding the qualitative sensitivity analysis to assess the robustness of the results, the overall conclusions were reassessed by excluding studies identified as having a high risk of bias. The study identified as high risk was Ito and Natsume [35], which was excluded from the sensitivity analysis. Exclusion of the high-risk study did not significantly alter overall results, suggesting the reliability of the review conclusions. Although the conclusions were more robust based on studies with low and moderate risk of bias, no articles were excluded to ensure a comprehensive understanding, despite introducing certain uncertainties.

From the articles included in this review, 146 characteristics associated with the ethical competency of nurse managers were identified. After applying the methodological process, 14 different characteristics were obtained (Table 1).

The characteristics most cited in the literature were ethical leadership (20.55%), ethical decision-making and conflict resolution (11.64%), organizational wellbeing and ethical culture (8.90%), comprehensive ethical management in healthcare (8.22%), and professional development and ethical promotion in nursing (8.22%). Therefore, the importance of leading with integrity and ethical principles and promoting an environment of trust and respect becomes evident. In addition, the need to integrate ethical principles into decision-making and organizational management to improve the quality of care and wellbeing of staff emerges. Continuing ethics education is also emphasized to prepare nurse managers to face ethical challenges in their daily practice.

This review identifies several ethical problems faced by nurse managers. These problems were systematically categorized and their frequencies were recorded (Table 2).

The most frequent ethical problem was the conflict between personal values and organizational policies. This highlights the constant struggle that nurse managers face in harmonizing their personal ethical convictions with the demands of their organizations. In addition, the management of limited resources, their allocation, and the pressure to compromise ethical values were also significant problems, indicating the complex environment in which nurse managers perform their roles. These issues underscore the need for strong ethical leadership and robust support systems to address ethical challenges effectively.

The review identified various consequences of the ethical problems faced by nurse managers. These consequences were systematically categorized and their frequencies were recorded.  Table 3 summarizes the primary ethical consequences reported in the literature.

The most frequently reported ethical consequences were decreased morale and job satisfaction among nursing staff and increased stress, burnout, and ethical distress (21.54%). These issues have a significant impact on the mental health and job satisfaction of the nursing staff. In addition, compromised patient care quality and safety (18.46%) and persistent ethical dilemmas leading to moral distress (16.92%) highlight the direct effects on patient outcomes and ethical climate within healthcare settings. These findings underscore the need for effective ethical leadership and support systems to mitigate adverse consequences and promote a healthier work environment.

This review identified several ethical principles that are frequently applied by nurse managers in their roles. These principles were systematically categorized, and their frequencies were recorded. The following table summarizes the primary ethical principles and their applications in nursing management, as reported in the literature (Table 4).

The ethical principles most frequently applied by nurse managers included autonomy, beneficence, nonmaleficence, justice, confidentiality, and integrity, each with 14 occurrences (20.59%). These principles highlight the commitment of nurse managers to empower their staff, ensure patient and staff welfare, and maintain high ethical standards. Consistent application of these principles fosters a fair and supportive work environment, promotes transparency, and improves overall quality of care. In addition, the principles of respect, communication, and role modeling, though less frequently, play crucial roles in maintaining a positive ethical climate and effective team dynamics within healthcare settings.

## 4. Discussion

This systematic review aimed to identify and describe the characteristics of nurses' competency in ethical principles. A total of 146 characteristics were identified and analyzed, which, after applying the methodological classification process, resulted in 14 distinct characteristics. An attempt was also made to contextualize this competency within the nurse manager environment.

Research highlights ethical leadership, ethical decision-making, and conflict resolution, together with the promotion of an ethical culture, as the most important characteristics for the development of competency in ethical principles. Essex et al. [36] pointed out that ethical leadership is a fundamental element to develop an ethical climate in healthcare organizations. Similarly, different studies have shown that ethical leadership is positively correlated with job satisfaction and affective commitment of healthcare personnel [37, 38]. Leaders who demonstrate ethical behavior and integrity can foster a supportive work environment, thus reducing stress and burnout among staff [39, 40]. In addition, ethical leaders who engage in open communication and provide support can significantly mitigate the risk of emotional exhaustion and burnout [41].

In addition, the ability to make ethical decisions and resolve conflicts is essential to maintain organizational harmony and ensure fair and equitable practices [42, 43]. In addition, promoting the wellbeing of the organization and its staff and fostering a culture of continuous professional development are vital to maintaining ethical practices in nursing leadership, as these actions significantly improve team cohesion and overall wellbeing [44, 45]. Mannion and Davies [46] emphasized that effective management includes respect for people, a fair working environment, the promotion of ethical practices, and quality patient care. Furthermore, the results of this research add evidence to studies that highlight how continuing education is the key to maintaining high ethical standards in nurse management [47, 48].

The ethical issues identified include conflicts between personal values and organizational policies, management of limited resources and their allocation, and pressures to compromise ethical values as the most frequently cited problems. These problems highlight the uncertain and often contradictory complexities in nurse managers' roles [49, 50]. In this regard, they must work to keep their personal ethical convictions aligned with the demands of the organization, potentially causing significant moral distress [51]. In addition, limited resource allocation and pressure to compromise ethical values pose challenges and difficulties in making appropriate decisions [52]. Therefore, ethical leadership and a culture based on transparency in decision-making are critical [53].

In relation to ethical issues, this study identified several consequences related to ethical issues faced by nurse managers. The lowest levels of morale and job satisfaction, along with increased stress, burnout, and ethical distress, were the most prevalent. These results are consistent with those of studies that highlight how ethical conflicts significantly affect mental health and job satisfaction of the nursing team [54, 55]. In addition, these problems can weaken trust and team cohesion [56, 57], raising concerns because they result in increased turnover and nurse attrition rates [58].

From the review, the key principles most frequently applied by nurse managers were identified, including autonomy, beneficence, nonmaleficence, fairness, confidentiality, and integrity. The consistent application of these ethical principles promotes a fair and supportive work environment [59]. In this sense, different research points on autonomy and beneficence are crucial for promoting independent decision-making [60]. Similarly, a nurse manager who incorporates these ethical principles into his functional role can create a more ethical and supportive work environment, thus improving patient outcomes and staff satisfaction [44, 61].

### 4.1. Limitations

The main limitation identified in this review relates to contextual factors specific to each healthcare setting, such as cultural, organizational, and regional differences. These factors can significantly influence the applicability and effectiveness of ethical principles in different contexts and their omission may limit the generalizability of the findings to diverse healthcare settings. This limitation was minimized through the methodological process of extracting characteristics and analyzing problems and their consequences, in which each extracted factor was analyzed and described independently of the contextual factors from which it originated.

### 4.2. Future Lines of Research

Future research should explore in depth the relationship between ethical principles in different contexts, with the aim of showing how cultural, organizational, and environmental differences influence the way ethical decisions are made and ethical leadership is exercised. It is also essential to conduct studies that evaluate the long-term improvement of healthcare environments when nurse managers have a high level of competency in ethical principles.

## 5. Conclusions

To achieve the adequate development of ethical principles, it is essential that nurse managers develop characteristics such as ethical leadership, ethical decision-making, and conflict resolution, together with the promotion of an ethical culture. Ethical leadership emerges as a fundamental characteristic that fosters an ethical climate in healthcare organizations, contributing significantly to the satisfaction of the job and affective commitment of healthcare personnel, thus reducing stress and burnout among staff. The ability to make ethical decisions and resolve conflicts is essential to maintaining organizational harmony and ensuring fair and equitable practices. In addition, promoting the wellbeing of the organization and its staff and fostering a culture of continuous professional development are the key to maintaining ethical practices.

The ethical issues faced by nurse managers indicate the uncertain and often contradictory complexities in which they must perform their roles. The need to align personal ethical convictions with the demands of the organization can cause significant moral distress. Establishing an open and transparent culture in an organization is essential to avoid these problems. These problems result in various consequences, such as moral distress, job dissatisfaction, increased stress, and burnout, which ultimately erode trust and team cohesion, impacting the quality of care. Therefore, nurse managers need to develop various characteristics that make up the competency of ethical principles.

The ethical principles most frequently used by nurse managers were autonomy, beneficence, nonmaleficence, justice, confidentiality, and integrity. The consistent application of these principles will be responsible for promoting an organizational culture of fairness by increasing transparency in decision making, which will result in improved quality of care.

## 6. Implications for the Nursing Management

The development of ethical leadership and the ability to make ethical decisions are fundamental characteristics of ethical principles competency, as they facilitate the achievement of an adequate work environment and reduce burnout and emotional overload. Therefore, nurse managers should prioritize the development of these characteristics to address the ethical dilemmas faced on a daily basis. This research provides essential knowledge for the future in which technology and Artificial Intelligence will play significant roles in both nurse management and care management.

## Figures and Tables

**Table 1:** Clustering and frequency of characteristics.

Characteristics	Frequency	Description
Ethical leadership	30 (20.55%)	Focuses on leadership that integrates ethical principles with decision-making, promoting integrity and ethical standards across the organization.
Ethical decision-making and conflict resolution	17 (11.64%)	Enhances ethical decision-making and conflict resolution through structured deliberation, negotiation, and administrative alignment.
Organizational wellbeing and ethical culture	13 (8.90%)	Foster a healthy organizational culture focused on ethical practices and employee wellbeing.
Comprehensive ethical management in healthcare	12 (8.22%)	Focuses on comprehensive ethical oversight, encompassing respectful treatment, resource management, and adherence to guidelines.
Professional development and ethical promotion in nursing	12 (8.22%)	Emphasizes ongoing education, professional empowerment, and ethical practice enhancement in nursing.
Communication and staff appreciation in healthcare	12 (8.22%)	Enhances staff morale and efficiency through effective communication and appreciation initiatives.
Ethical routines in care	9 (6.16%)	Emphasizes consistent, high-quality care through established ethical routines and patient-centered practices.
Transparent and ethical decision-making	9 (6.16%)	Promotes transparent processes and ethical clarity in decision-making across healthcare operations.
Ethical support and crisis management in healthcare	8 (5.48%)	Provides ethical guidance and support during crises, ensuring healthcare integrity under stress.
Resolution of problems and wishes	6 (4.11%)	Facilitates effective problem resolution and fulfills staff needs, enhancing workplace harmony.
Advocacy for patient rights and dignity	5 (3.42%)	Dedicated to upholding and defending patients' rights and dignity within healthcare settings.
Strategic ethical policy development	5 (3.42%)	Focuses on the creation and implementation of strategic ethical policies to guide healthcare practices.
Adherence to ethical rules	4 (2.74%)	Strict enforcement of ethical guidelines to ensure just and fair practices in healthcare.
Alleviation of suffering	4 (2.74%)	Promote compassionate practices and care to alleviate suffering in healthcare settings.

**Table 2:** Nurse manager ethical problems.

Ethical problem	Frequency
Conflicts between personal values and organizational policies	10 (22.22%)
Management of conflicts and interpersonal tensions	7 (15.56%)
Pressures to compromise ethical values	8 (17.78%)
Management of limited resources and resource allocation	9 (20.00%)
Lack of support and recognition for nursing staff	6 (13.33%)
Communication barriers and lack of managerial support	5 (11.11%)
Frustration and moral distress due to contrary organizational priorities	4 (8.89%)
Issues with a consistent application of ethical standards	3 (6.67%)
Challenges in integrating personal and professional ethics	2 (4.44%)

**Table 3:** Ethical consequences and their frequency.

Ethical consequence	Frequency
Decreased morale and job satisfaction among nursing staff	14 (21.54%)
Increased stress, burnout, and ethical distress	14 (21.54%)
Compromised patient care quality and safety	12 (18.46%)
Persistent ethical dilemmas leading to moral distress	11 (16.92%)
Erosion of trust and team cohesion	10 (15.38%)
Increased turnover rates and intention to leave	9 (13.85%)
Communication breakdowns and poor management support	7 (10.77%)
Challenges in ethical decision-making and management	6 (9.23%)
Negative impact on psychological wellbeing and performance	5 (7.69%)

**Table 4:** Ethical principles and their application in nursing management.

Ethical principles	Frequency	Application in nursing management by nurse managers
Autonomy	14 (20.59%)	Nurse managers empower nurses by promoting independent decision-making within ethical guidelines, enhancing professional judgment.
Beneficence	14 (20.59%)	Actions taken by nurse managers aim to benefit patients and staff by optimizing care quality and resource allocation.
Nonmaleficence	14 (20.59%)	Nurse managers work to prevent harm by managing work conditions and responsibilities to safeguard staff welfare and patient safety.
Justice	14 (20.59%)	Equitable treatment is ensured through fair policies on task assignments, promotions, and resource distribution.
Confidentiality	14 (20.59%)	The protection of sensitive information is upheld through strict confidentiality protocols for patients and staff.
Integrity	14 (20.59%)	Nurse managers maintain high ethical standards, adhere to professional codes, and make decisions with moral integrity under all conditions.
Respect	5 (7.35%)	Respect is maintained in interactions, creating a supportive work environment where ethical conflicts are effectively managed.
Communication	6 (8.82%)	Open and effective communication is promoted to ensure transparency and understanding across healthcare settings.
Role modeling	5 (7.35%)	Nurse managers serve as ethical role models, influencing nursing staff through their conduct and leadership in ethical decision-making.
Empathy	4 (5.88%)	Demonstrating understanding and compassion in interactions, which strengthens relational connections and support within the team.
Supportiveness	4 (5.88%)	Providing ongoing support and resources to assist nursing staff in overcoming professional challenges and achieving objectives.
Crisis management and ethical climate maintenance	3 (4.41%)	Effective leadership during ethical crises is crucial, as is the continuous effort to enhance the ethical climate of the healthcare environment.
Conflict resolution and transparency	3 (4.41%)	Active mediation in conflicts ensures fair resolutions and open communication fosters transparency and trust.
Professional development and ethics training	3 (4.41%)	Promoting continuous professional and ethical development through training and educational programs.

## Data Availability

The data that support the findings of this study are available from the corresponding author upon reasonable request.

## Supporting Information

Supporting file 1: Table S1: Article search strategy; Table S2: Articles included in the review; Table S3: Quality appraisal of quantitative papers; Table S4: Quality appraisal of qualitative papers; Table S5: Risk of bias assessment for included studies. References tables S1–S5.
