# Supplementary material for: Characteristics of the Competency Ethical Principles for the Nurse Manager: A Systematic Review
Source: J Nurs Manag. 2025 Feb 26;2025:2575609. doi: 10.1155/jonm/2575609 (PMC11985233; doi:10.1155/jonm/2575609)
Supplement: Supporting Information — Additional supporting information can be found online in the Supporting Information section. [file 2575609.f1.docx]

**Supplementary File 1**

**Table S1.** Search strategy

| **Database** 2011-2021 | | Search Terms |
| --- | --- | --- |
| **WOS** | TI=(“**Ethic* princip*** ” OR ethic* OR “ethic* valu*”) AND TI=("nurs* manage*" OR "nurse supervisor" OR "nursing program manager" OR "nurse unit manager" OR "chief nurse executive" OR "nurs* administrat*" OR "director of nurs*" OR "head nurs*" OR "frontline manager" OR "nurs* director" OR "nurs* executiv*") AND TS=("head nurs*" OR "frontline manager" OR "nurs* director" OR "nurs* manag*" OR "first line nurs* manage*" OR "nurse supervisor" OR "nursing program manager" OR "nurse unit manager" OR "chief nurse executive" OR "nurs* administrat*" OR "director of nurs*" OR "nurs* executiv*") AND TS=(“**Ethic* princip*** ” OR ethic* OR “ethic* valu*”) | |
| **SCOPUS** | TITLE-ABS-KEY("nurs* manage*" OR "nurse supervisor" OR "nursing program manager" OR "nurse unit manager" OR "chief nurse executive" OR "nurs* administrat*" OR "director of nurs*" OR "head nurs*" OR "frontline manager" OR "nurs* director" OR "nurs* executiv*") AND TITLE-ABS-KEY("Ethic* princip*" OR ethic* OR "ethic* valu*") | |
| **PUBMED** | ("Ethic* princip*" OR ethic* OR "ethic*") AND (("Nurse Administrators"[Majr] OR "Nursing, Supervisory"[Majr]) | |

**Table S2.** Articles included in the review

| Authors (year) | Design | Participants (n) and Population | Variable(s) | Application in Nursing | Main Findings |
| --- | --- | --- | --- | --- | --- |
| Aitamaa et al. (2016) [62] | Descriptive, qualitative | 9 nurse managers from primary and specialized healthcare | Ethical problems in management, ethical dilemmas | Ethical decision-making in nursing management | Identified ethical problems categorized into conflicts, lack of appreciation, disregard of problems, and experienced inadequacy. Enhanced understanding of ethical challenges in nursing management. |
| Aitamaa et al. (2019) [63] | Cross-sectional survey | 214 nurse managers from ward, middle, and strategic management | Ethical problems related to patients, staff, and organization | Management of ethical issues in nursing | Frequent ethical problems related to staff and organization; significant difficulties associated with organization-related ethical problems. |
| Aitamaa et al. et al. 2019) [64] | Cross-sectional survey | 214 nurse managers from ward, middle, and strategic management | Ethical problems related to patients, staff, and organization | Management of ethical issues in nursing | Discussions and personal values are the most used methods for solving ethical problems, with discussions being the most useful. |
| Alan et al. (2022) [65] | Descriptive, correlational, cross-sectional | 285 nurses from public and private hospitals in Istanbul | Ethical leadership and conflict management strategies | Enhancing nurse managers' conflict management | Ethical leadership in nurse managers is positively associated with collaborative conflict management strategies. |
| Barkhordari et al. (2016) [67] | Qualitative, content analysis | 14 nurse leaders and faculty members from various levels of management | Ethical competency in cultural domains | Enhancing ethical competency in nurse leaders | Identified ethical competencies in nurse leaders which include empathetic interactions, ethical behavior, and exalted manners. |
| Barkhordari et al. (2017b) [69] | Qualitative, content analysis | 14 nursing leaders and educators from Iran | Obstacles and problems of ethical leadership in nursing | Enhancing ethical leadership | Identified ethical, cultural, and managerial obstacles impeding ethical leadership in the nursing profession. |
| Cao et al. (2021) [70] | Qualitative, content analysis | 19 new frontline nurse managers from a hospital in Shandong, China | Ethical challenges of new nurse managers | Management of ethical challenges | New nurse managers face ethical challenges such as trust crises and conflicts with administration. |
| Cunha et al. (2023) [71] | Qualitative, content analysis | 19 nurse managers at a university hospital in Brazil | Ethical problems experienced by nurse managers during COVID-19 | Managing ethical issues in nursing | Identified ethical challenges such as misinformation, staff management issues, and patient-related conflicts during COVID-19. |
| Devik et al. (2020) [72] | Qualitative, content analysis | 10 nurse managers in nursing homes and home care services in Norway | Ethical support in everyday nursing management | Support for ethical competence in nursing | Nurse managers need to enhance support for ethical competence; everyday ethics includes handling ethical challenges effectively. |
| Ganz et al. (2014) [73] | Cross-sectional survey | Middle nurse managers from four hospitals in Israel | Ethical dilemmas and moral distress | Enhancing ethical environments | Nurse middle managers experience distinct ethical dilemmas and moral distress, differing from staff nurses. |
| Hognestad Haaland, et al. (2021) [74] | Cross-sectional survey | 2,946 registered nurses in Norway | Supervisor support, ethical dilemmas, meaning of work, intention to leave | Reduce turnover, enhance job satisfaction | Supervisor support and ethical dilemmas influence nurses' intention to leave mediated by meaning of work. |
| Ito and Natsume (2015) [35] | Anonymous questionnaires and semi-structured interviews | 4 chief nurses from a nursing ethics workshop in Japan | Ethical dilemmas in nursing management | Ethical decision-making in nursing management | Chief nurses face unique ethical dilemmas related to patient dignity and management, often without resolution. |
| Khosravani et al. (2024) ) [75] | Phenomenological study | 20 nurse managers in Arak, Iran | Ethical challenges and solutions in management | Management of ethical challenges | Nurse managers face significant ethical challenges related to personnel, organizational, and resource issues, and identify practical organizational, interpersonal, and educational solutions. |
| Laukkanen et al. (2016) ) [76] | Cross-sectional survey | 122 nurse managers in Finland | Ethical activities: development, influence, research, identification, and resolution of ethical issues | Ethical management and problem-solving | Nurse managers identify many ethical issues, but less actively engage in developing ethical knowledge or conducting research. |
| Laukkanen et al. (2015) ) [21] | Cross-sectional survey | 122 nurse managers in Finnish healthcare organizations | Solving work-related ethical problems | Ethical problem-solving in nursing management | Nurse managers use a variety of non-systematic activities to solve ethical problems, including discussion, cooperation, and work organization. |
| (Barkhordari-(2017a) [68] | Qualitative study | 14 nursing faculty members and nursing managers in Iran | Ethical leadership in nursing | Leadership and ethical climate in nursing | Ethical leadership leads to all-inclusive satisfaction (inner peace, employee and patient satisfaction) and increased productivity through improved services and ethical behavior inspiration. |
| Salmela et al. (2016) ) [78] | Mixed Methods | 375 staff members from eight hospital units in western Finland | Ethical guidelines and norms, views on evidence-based care | Ethical management and leadership | The study focused on ethical leadership in nursing, emphasizing the importance of nurturing a caring and ethically sustainable culture in healthcare settings. The responses highlighted the value placed on professional competence, the common good, and the alleviation of suffering among other ethical dimensions. |
| Whitehead et al. (2021) ) [79] | Qualitative descriptive study | 19 nurse managers from 5 healthcare institutions in Virginia | Moral distress among nurse managers | Managing moral distress in nursing | Nurse managers experience moral distress primarily when their ability to ensure effective unit operation is compromised by organizational directives. |
| Nejat et al. (2023) ) [44] | Qualitative phenomenological study | 20 nurse managers from hospitals in Arak, Iran | Managerial ethics | Ethics in nursing management | Identified five themes of managerial ethics: professional ethics, people-oriented management, professional empowerment, excellencism, and patient-centered care. |
| Yu et al. (2024) [80] | Quantitative, cross-sectional | 309 head nurses in Hunan, China | Ethical climate, moral resilience, and ethical competence in nursing | Leadership and management in nursing | Moral resilience mediates the relationship between ethical climate and ethical competence in head nurses. |
| Asgari, et al. (2019) [66] | Qualitative, content analysis | 10 nurse managers and 7 nurses from hospitals in Iran | Ethical competency of nurse managers | Enhancing ethical leadership in nursing management | Identified four main attributes of ethical competency: flexibility, approachability, observance of morality, and practical modeling. |
| Roshanzadeh (2020) ) [77] | Qualitative, content analysis | 19 nurse managers from Tehran hospitals | Moral sensitivity in decision-making | Ethical decision-making in nursing management | Moral sensitivity influences nurse managers to make excellent decisions by enhancing their assertiveness, commitment, and insight. |

**Table S3.** Quality Appraisal of Quantitative papers

| Citations | Q1 | Q2 | Q3 | Q4 | Q5 | Q6 | Q7 | Q8 | Total score | Share of answers yes (%) |
| --- | --- | --- | --- | --- | --- | --- | --- | --- | --- | --- |
| Aitamaa et al. (2019) [63] | Yes | Yes | Yes | Yes | Yes | Yes | Yes | Yes | 8/8 | 100% |
| Aitamaa et al. et al. 2019) [64] | Yes | Yes | Yes | Yes | Yes | Yes | Yes | Yes | 8/8 | 100% |
| Alan et al. (2022) [65] | Yes | Yes | Yes | Yes | Yes | Yes | Yes | Yes | 8/8 | 100% |
| (Hognestad Haaland, et al. (2021) [74] | Yes | Yes | Yes | Yes | Yes | Yes | Yes | Yes | 8/8 | 100% |
| Ito and Natsume (2015) [35] | Yes | Yes | Unclear | Yes | Yes | Yes | Yes | Yes | 7/8 | 87.5% |
| Khosravani et al. (2024) [75] | Yes | Yes | Yes | Yes | Yes | Yes | Yes | Unclear | 7/8 | 87.5% |
| Laukkanen et al. (2016) [76] | Yes | Yes | Yes | Yes | Yes | Yes | Yes | Yes | 8/8 | 100% |
| Laukkanen et al. (2015) [21] | Yes | Yes | Yes | Yes | Yes | Yes | Yes | Yes | 8/8 | 100% |
| Yu et al. (2024) [80] | Yes | Yes | Yes | Yes | Yes | Yes | Yes | Yes | 8/8 | 100% |
| Salmela et al. (2016) [78] | Yes | Yes | Yes | Yes | Yes | Yes | Yes | Yes | 8/8 | 100% |

**Table S4.** Quality Appraisal of qualitative papers

| Citations | Q1 | Q2 | Q3 | Q4 | Q5 | Q6 | Q7 | Q8 | Q9 | Q10 | Total score | Share of answers yes (%) |
| --- | --- | --- | --- | --- | --- | --- | --- | --- | --- | --- | --- | --- |
| Aitamaa et al. (2016) [62] | Descriptive, qualitative | Yes | Yes | Yes | Yes | No | No | Yes | Yes | Yes | 8/10 | 80% |
| Barkhordari et al. (2016) [67] | Yes | Yes | Yes | Yes | Yes | Yes | Yes | Yes | Yes | Yes | 10/10 | 100% |
| Barkhordari et al. (2017a) [68] | Yes | Yes | Yes | Yes | Yes | Yes | Yes | Yes | Yes | Yes | 10/10 | 100% |
| Barkhordari et al. (2017b) [69] | Yes | Yes | Yes | Yes | Yes | Yes | Yes | Yes | Yes | Yes | 10/10 | 100% |
| Cao et al. (2021) [70] | Yes | Yes | Yes | Yes | Yes | Yes | Yes | Yes | Yes | Yes | 10/10 | 100% |
| Cunha et al. (2023) [71] | Yes | Yes | Yes | Yes | Yes | Yes | Yes | Yes | Yes | Yes | 10/10 | 100% |
| Devik et al. (2020) [72] | Yes | Yes | Yes | Yes | Yes | Yes | Yes | Yes | Yes | Yes | 10/10 | 100% |
| Ganz et al. (2014) [73] | Yes | Yes | Yes | Yes | Yes | Yes | Yes | Yes | Yes | Yes | 10/10 | 100% |
| (Whitehead et al. (2021) [79] | Yes | Yes | Yes | Yes | Yes | Unclear | Yes | Yes | Yes | Yes | 9/10 | 90% |
| Nejat et al. (2023) [44] | Yes | Yes | Yes | Yes | Yes | Unclear | Yes | Yes | Yes | Yes | 9/10 | 90% |
| (Barkhordari-(2017a) [68] | Yes | Yes | Yes | Yes | Yes | Yes | Yes | Yes | Yes | Yes | 10/10 | 100% |
| Asgari, et al. (2019) [66] | Yes | Yes | Yes | Yes | Yes | Yes | Yes | Yes | Yes | Yes | 10/10 | 100% |
| Roshanzadeh (2020) [77] | Yes | Yes | Yes | Yes | Yes | Yes | Yes | Yes | Yes | Yes | 10/10 | 100% |

**Table S5**. Risk of Bias Assessment for Included Studies

| Author (Year) | Study Design | Sample Size | Bias Evaluation | Risk of Bias |
| --- | --- | --- | --- | --- |
| Aitamaa et al. (2016) [62] | Descriptive, qualitative | 9 | High methodological quality in data collection and analysis | Low |
| Aitamaa et al. (2019) [63] | Cross-sectional survey | 214 | Good practices in sampling and analysis, limited control of confounding factors | Moderate |
| Aitamaa et al. et al. 2019) [64] | Cross-sectional survey | 214 | Good control of variables, but potential limitations in data analysis | Moderate |
| Alan et al. (2022) [65] | Descriptive, correlational, cross-sectional | 285 | Adequate methods with potential biases in data analysis | Moderate |
| Barkhordari et al. (2016) [67] | Qualitative content analysis | 14 | Good practices in interviews and thematic analysis, limited sample size | Moderate |
| Barkhordari et al. (2017b) [69] | Qualitative content analysis | 14 | Good practices in interviews and thematic analysis, limited sample size | Moderate |
| Cao et al. (2021) [70] | Qualitative content analysis | 19 | High quality in data collection and analysis, limited sample size | Moderate |
| Cunha et al. (2023) [71] | Qualitative content analysis | 19 | High quality in data collection and analysis, limited sample size | Moderate |
| Devik et al. (2020) [72] | Qualitative content analysis | 10 | Good practices in interviews and thematic analysis, limited sample size | Moderate |
| Ganz et al. (2014) [73] | Cross-sectional survey | 4 hospitals | Good control of variables, but potential limitations in data analysis | Moderate |
| Hognestad Haaland, et al. (2021) [74] | Cross-sectional survey | 2946 | Large sample size with appropriate methods, limited control of confounding variables | Moderate |
| Ito and Natsume (2015) [35] | Qualitative study | 4 | High quality in data collection and ethical considerations, very small sample size | High |
| Khosravani et al. (2024) ) [75] | Phenomenological study | 20 | High quality in data collection and analysis, limited sample size | Moderate |
| Laukkanen et al. (2016) ) [76] | Cross-sectional survey | 122 | Good practices in sampling and analysis, limited control of confounding factors | Moderate |
| Laukkanen et al. (2015) ) [21] | Cross-sectional survey | 122 | Adequate methods with potential biases in data analysis | Moderate |
| (Barkhordari-(2017a) [69] | Qualitative study | 14 | High quality in data collection and thematic analysis, limited sample size | Moderate |
| Salmela et al. (2016) ) [78] | Mixed Methods | 375 | High quality in both qualitative and quantitative data collection, some limitations in integration | Moderate |
| Whitehead et al. (2021) ) [79] | Qualitative descriptive study | 19 | Good practices in data collection and thematic analysis, limited sample size | Moderate |
| Nejat et al. (2023) ) [44] | Qualitative phenomenological study | 20 | High quality in data collection and thematic analysis, limited sample size | Moderate |
| Yu et al. (2024) [80] | Quantitative, cross-sectional | 309 | Solid evaluation with some limitations in variable control | Moderate |
| Asgari, et al. (2019) [66] | Qualitative content analysis | 17 | High quality in data collection and thematic analysis, limited sample size | Moderate |
| Roshanzadeh (2020) ) [77] | Qualitative content analysis | 19 | Good practices in data collection and analysis, sample size limitations | Moderate |

**References tables S1 to S5**

[21] L. Laukkanen, R. Suhonen, H. Leino-Kilpi, “Solving work-related ethical problems: The activities of nurse managers,” Nurs Ethics, vol. 23, no. 8, pp. 838–850, 2015.

[35] C. Ito, M. Natsume, “Ethical Dilemmas Facing Chief Nurses in Japan: A Pilot Study,” Nursing Ethics, vol. 23, no. 4, pp. 432–441, 2015.

[44] N. Nejat, S. Zand, M. Taheri, M. Khosravani, “Understanding lived experiences of nurse managers about managerial ethics,” *Nurs Ethics*, vol. 30, no. 2, pp. 162–179, 2023.

[62] E. Aitamaa, H. Leino-Kilpi, S. Iltanen, R. Suhonen, “Ethical Problems in Nursing Management: The Views of Nurse Managers,” Nurs Ethics, vol. 23, no. 6, pp. 646–658, 2016.

[63] E. Aitamaa, R. Suhonen, S. Iltanen, P. Puukka, H. Leino-Kilpi, “Ethical Problems in Nursing Management: Frequency and Difficulty of the Problems,” Health Care Manage Rev, vol. 46, no. 1, pp. 25–34, 2019.

[64] E. Aitamaa, R. Suhonen, P. Puukka, H. Leino-Kilpi, “Ethical Problems in Nursing Management - a Cross-Sectional Survey about Solving Problems,” BMC Health Serv Res, vol. 19, no. 1, pp. 417, 2019.

[65] H. Alan, D. Gül, Ü. Baykal, “The Relationship between the Conflict Management Strategies and Ethical Leadership Behaviours of Nurse Managers Perceived by Nurses,” J Nurs Manag, vol. 30, no. 7, pp. 2370–2378, 2022.

[66] N. Asgari, H. Navipour, E. Mohammadi, “Ethical Competency of Nurse Managers - Content Analysis,” J Evol Med Dent Sci, vol. 8, no. 51, pp. 3873–3877, 2019.

[67] M. Barkhordari-Sharifabad, T. Ashktorab, F. Atashzadeh-Shoorideh, “Ethical Competency of Nurse Leaders: A Qualitative Study,” Nurs Ethics, vol. 25, no. 1, pp. 20–36, 2016.

[68] M. Barkhordari-Sharifabad, T. Ashktorab, F. Atashzadeh-Shoorideh, “Ethical Leadership Outcomes in Nursing: A Qualitative Study,” Nurs Ethics, vol. 25, no. 8, pp. 1051–1063, 2017a.

[69] M. Barkhordari-Sharifabad, T. Ashktorab, F. Atashzadeh-Shoorideh, “Obstacles and Problems of Ethical Leadership from the Perspective of Nursing Leaders: A Qualitative Content Analysis,” J Med Ethics Hist Med, vol. 10, 2017b.

[70] L. Cao, J. Bian, J. Xiao, Y. Jia, “Trust Crisis: New Frontline Nurse Managers’ Ethical Experience in Their Units,” J Nurs Scholarsh, vol. 54, no. 2, pp. 250–257, 2021.

[71] S. G. S. Cunha, S. Deodato, M. J. M. Brito, “Ethical Problems Experienced by Nurse Managers in the Pandemic of COVID-19 in a University Hospital,” Rev Rene, vol. 24, 2023.

[72] S. A. Devik, H. Munkeby, M. Finnanger, A. Moe, “Nurse Managers’ Perspectives on Working with Everyday Ethics in Long-Term Care,” Nurs Ethics, vol. 27, no. 8, pp. 1669–1680, 2020.

[73] F. D. Ganz, N. Wagner, O. Toren, “Nurse Middle Manager Ethical Dilemmas and Moral Distress,” Nurs Ethics, vol. 22, no. 1, pp. 43–51, 2014.

[74] G. Hognestad Haaland, E. Olsen, A. Mikkelsen, “The Association between Supervisor Support and Ethical Dilemmas on Nurses’ Intention to Leave: The Mediating Role of the Meaning of Work,” J Nurs Manag, vol. 29, no. 2, pp. 286–293, 2021.

[75] M. Khosravani, M. Taheri, N. Amini, N. Babaeian, S. Lak, N. Nejat, “Explaining Ethical Challenges and Practical Solutions from a Nursing Managements’ Perspective: A Phenomenological Study,” J Fam Med Prim Care, vol. 13, no. 2, pp. 681–690, 2024.

[76] L. Laukkanen, H. Leino-Kilpi, R. Suhonen, “Ethical Activity Profile of Nurse Managers,” J Nurs Manag, vol. 24, no. 4, pp. 483–491, 2016.

[77] M. Roshanzadeh, Z. Vanaki, A. Sadooghiasl, “Sensitivity in Ethical Decision-Making: The Experiences of Nurse Managers,” Nurs Ethics, vol. 27, no. 5, pp. 1174–1186, 2020.

[78] S. Salmela, C. Koskinen, K. Eriksson, “Nurse Leaders as Managers of Ethically Sustainable Caring Cultures,” J Adv Nurs, vol. 73, no. 4, pp. 871–882, 2016.

[79] P. B. Whitehead, K. F. Carter, J. S. Garber, B. Epstein, “The Nurse Manager’s Experience of Moral Distress,” J Nurs Adm, vol. 51, no. 6, pp. 334–339, 2021.

[80] Q. Yu, C. Huang, J. Yan, L. Yue, Y. Tian, J. Yang, X. Li, Y. Li, Y. Qin, “Ethical Climate, Moral Resilience, and Ethical Competence of Head Nurses,” Nurs Ethics, 2024.
